# Creating zero-field skyrmions in exchange-biased multilayers through X-ray illumination

**DOI:** 10.1038/s41467-020-14769-0

**Published:** 2020-02-19

**Authors:** Yao Guang, Iuliia Bykova, Yizhou Liu, Guoqiang Yu, Eberhard Goering, Markus Weigand, Joachim Gräfe, Se Kwon Kim, Junwei Zhang, Hong Zhang, Zhengren Yan, Caihua Wan, Jiafeng Feng, Xiao Wang, Chenyang Guo, Hongxiang Wei, Yong Peng, Yaroslav Tserkovnyak, Xiufeng Han, Gisela Schütz

**Affiliations:** 1 Beijing National Laboratory for Condensed Matter Physics, Institute of Physics, Chinese Academy of Sciences, Beijing, 100190 China; 2 Center of Materials Science and Optoelectronics Engineering, University of Chinese Academy of Sciences, Beijing, 100049 China; 3 Songshan Lake Materials Laboratory, Dongguan, 523808 Guangdong China; 4 Max Planck Institute for Intelligent Systems, Heisenbergstraße 3, 70569 Stuttgart, Germany; 5 Department of Physics and Astronomy, University of California, Los Angeles, CA 90095 USA; 6 Department of Physics and Astronomy, University of Missouri, Columbia, MO 65211 USA; 7 Key Laboratory for Magnetism and Magnetic Materials of Ministry of Education, Lanzhou University, Lanzhou, 730000 People’s Republic of China

**Keywords:** Spintronics, Spintronics

## Abstract

Skyrmions, magnetic textures with topological stability, hold promises for high-density and energy-efficient information storage devices owing to their small size and low driving-current density. Precise creation of a single nanoscale skyrmion is a prerequisite to further understand the skyrmion physics and tailor skyrmion-based applications. Here, we demonstrate the creation of individual skyrmions at zero-field in an exchange-biased magnetic multilayer with exposure to soft X-rays. In particular, a single skyrmion with 100-nm size can be created at the desired position using a focused X-ray spot of sub-50-nm size. This single skyrmion creation is driven by the X-ray-induced modification of the antiferromagnetic order and the corresponding exchange bias. Furthermore, artificial skyrmion lattices with various arrangements can be patterned using X-ray. These results demonstrate the potential of accurate optical control of single skyrmion at sub-100 nm scale. We envision that X-ray could serve as a versatile tool for local manipulation of magnetic orders.

## Abstract (web summary)

Skyrmions are objects with whirled magnetization protected by their topology that can be created by different means, however, without control of their position. Here, the authors present a method exploiting x-rays to create skyrmions at the beam position allowing for creation of artificial skyrmion lattices.

## Introduction

Magnetic skyrmions are topologically protected spin textures arising in magnetic materials most commonly with the Dzyaloshinskii–Moriya interaction (DMI)^1,2^. Although first observed in B20-type bulk compounds^3,4^, which host the intrinsic DMI, skyrmions residing in magnetic thin films with tunable interfacial DMI are more applicable to practical applications^5–10^. Precise creation of a single nanoscale skyrmion is of vital importance for understanding and harnessing magnetic skyrmions. To date, the electrical creations of a single skyrmion have been experimentally demonstrated^5,11^, e.g., spin-polarized currents or electric fields can favor the flip of spins and induce the creation of a single skyrmion. By leveraging a non-uniform device geometry or a naturally formed defect, a current pulse can also generate a single skyrmion^7,12–14^. In addition to electrical means, optical methods can also offer an efficient way for manipulating magnetizations, with the advantages of ultrafast dynamics and flexibility in selecting the writing position^15^. Recent proposals have predicted that lasers can be used to create skyrmions via laser fields or local heating^16–18^. However, the spot size of conventional lasers is fundamentally limited by the light wavelengths to above several hundred nanometers. Thus these lasers can only create either single large-sized magnetic bubbles^15,16,19,20^ or groups of skyrmions^16,21,22^, while creation of single small skyrmions requires excitation light with a shorter wavelength.

Soft X-rays have been used for magnetic imaging^23^ thanks to the high spatial resolution associated with its short wavelengths of the order of a few nm and the existence of a strong magnetic contrast mechanism in the form of X-ray magnetic circular dichroism, which causes X-ray absorption to depend on the magnetization component parallel to the wave vector of incident light^24^. Although X-rays have been extensively used as a probe technique, their potential for manipulating magnetization is still largely unexplored.

In this work, we demonstrate that soft X-rays can create single skyrmions with 100-nm size at zero field and room temperature in an exchange-biased magnetic multilayer. We find that the X-ray can induce an unexpected exchange bias reorientation effect. Based on this effect, single skyrmions, skyrmion-track, and artificial skyrmion lattice are successfully created. The high spatial resolution of X-ray provides a unique optical tool for creating single small skyrmions. Furthermore, the created skyrmion-track and artificial skyrmion lattice can not only serve as a versatile platform for studying skyrmion physics and topological phenomena but may also lead to potential skyrmionic applications.

## Results

### Observation of the exchange bias reorientation effect

**Fig. 1:**
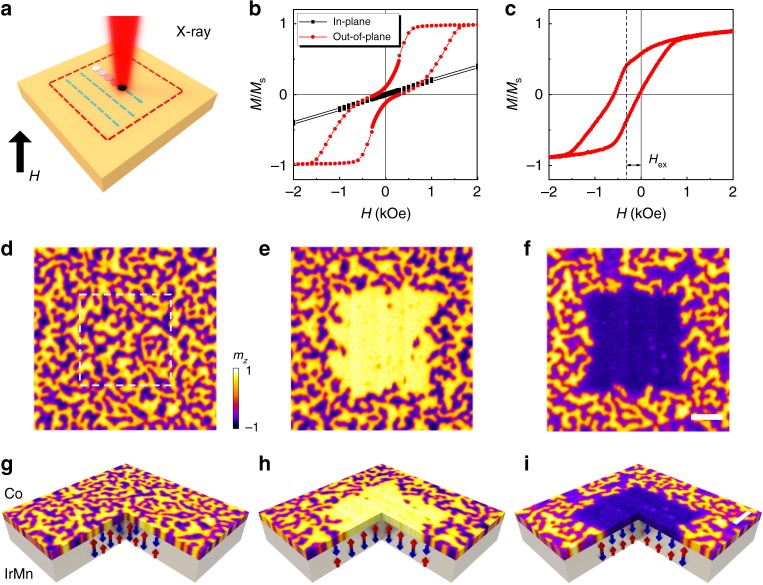
X-ray induced exchange bias reorientation effect. **a** Sketch of using synchrotron X-rays to introduce a uniform exchange bias through scanning a closed area (dashed lines) under a perpendicular magnetic field (*H*). The magnetic field indicated by the arrow is along the positive direction. **b** Out-of-plane (red) and in-plane (black) hysteresis loops for the as-grown sample. **c** Hysteresis loop at 300 K after field cooling process. The field cooling is performed from 400 K and with the field of 1.5 T. *H*_ex_ indicates the introduced exchange bias in the out-of-plane direction. **d** Initial magnetic domain pattern imaged by scanning transmission X-ray microscopy (STXM) at zero magnetic field for a sample with structure of Pt (5)/Co (0.6)/IrMn (2)/[Pt (2)/Co (0.6)/IrMn (2)]_11_/NiO (2) (thickness in nm). **e**, **f** Zero-field magnetic domain pattern (6 μm × 6 μm) after scanning the central area (3 × 3 μm^2^) using a circular X-ray under magnetic fields of 2000 Oe (**e**) and −2000 Oe (**f**). The scanning process induces a uniform exchange bias in the scanned area. The exchange bias direction is along the direction of the applied magnetic field. **g**–**i** Schematics of the three-quarter sectional cut of the STXM data and the corresponding exchange bias. The arrows in the IrMn layer schematically indicate the antiferromagnetically ordered net magnetization in the out-of-plane direction. The white scale bar in **f**, **i** is 1 μm.

The studied sample consists of Si_3_N_4_/Pt (5 nm)/Co (0.6 nm)/IrMn (5 nm)/[Pt (2 nm)/Co (0.6 nm)/IrMn (5 nm)]_11_/NiO (2 nm) (for details of the sample structure, see Supplementary Note 1). The sample shows a perpendicular magnetic anisotropy (see Fig. 1b and Supplementary Note 2). No exchange bias is observed in the out-of-plane *M*–*H* loop of the as-grown sample (Fig. 1b), because the interfacial uncompensated spins are not uniformly aligned in a single direction. The interfacial uncompensated spin can be realigned through a typical field-cooling process, resulting in the appearance of an exchange bias (Fig. 1c, also see Supplementary Note 2), which can enhance the stability for skyrmions at zero magnetic field^25^.

To characterize the detailed domain structure of the sample, an illumination process is carried out using a scanning transmission X-ray microscopy (STXM)^26^ by means of a step-by-step scanning, as illustrated in Fig. 1a. We first perform a scanning process over the studied as-grown sample at zero field with a dwell time of 5 ms. As shown in Fig. 1d, g, the sample exhibits a typical labyrinth domain pattern at the remnant state. When we scan the central area in Fig. 1d with a dwell time of 30 ms in the presence of a 2000 Oe saturation field (perpendicular to the film), an unexpected result is observed that the illuminated area (dashed square) becomes a single domain at zero magnetic field after the illumination, as shown in Fig. 1e, h. The magnetization direction of the central area can also be reoriented in the opposite direction by applying an opposite magnetic field during the illumination, as shown in Fig. 1f, i. These are in sharp contrast to the unaffected labyrinth pattern outside the illuminated central area.

Since the as-grown sample has randomly distributed local exchange bias, it might be possible that a uniform exchange bias is introduced by the X-ray illumination inside a magnetic bias field. In this case, if the magnitude of this exchange bias is close to the saturation field of the sample, it can overcome the dipole–dipole interaction (which favors a labyrinth domain structure) and maintain a single domain pattern at zero magnetic field. Such formation of a uniform exchange bias is confirmed by the local hysteresis loop measurements of the illuminated central area (see Supplementary Note 3). Similar exchange bias reorientation effects have also been observed in a series of samples (see Supplementary Note 4). These observations imply that the antiferromagnetic correlations of IrMn are disturbed during the X-ray illumination and are realigned according to the adjacent Co magnetization direction after the illumination, resulting in the formation of a uniform exchange bias within the illumination region.

**Fig. 2:**
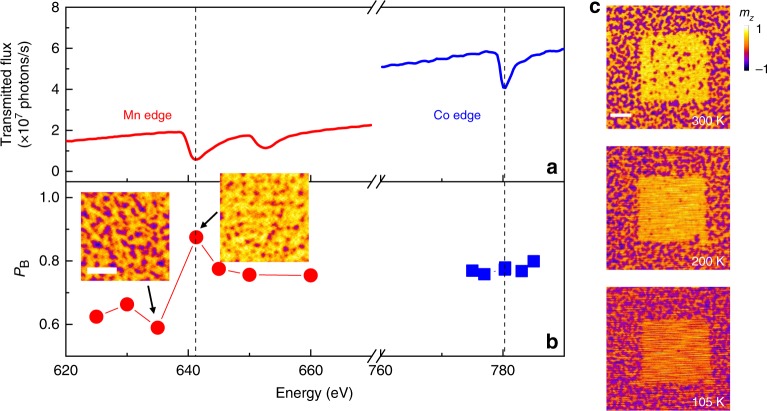
Photon energy and temperature dependence of the exchange bias reorientation effect. **a** Detected photon flux after transmission through the sample as a function of photon energy. The flux is calculated based on the detected photons within 2 s by the detector behind the sample. **b** Percentage of the bright area *P*_B_ = *S*(*M*_↑_)/*S*(*M*_↑_ + *M*_↓_) as a function of photon energy. *S*(*M*_↑_) and *S*(*M*_↓_) represent the area for magnetization point up (bright) and down (dark), respectively. The red dots (blue square) show the effect of X-ray illumination on the energy near the Mn (Co) edge. The measurements were done for a sample with 0.4-nm-thick Co layer. The scale bar is 1 μm. **c** Zero-field magnetic domain pattern (6 μm × 6 μm) after scanning the central area (3 × 3 μm^2^) using X-ray under a magnetic field of 2000 Oe at 300, 200, and 105 K. The dwell time used for scanning each pixel is 3 ms. The scale bar is 1 μm.

To further confirm that the exchange bias reorientation is due to the interaction between the X-ray and IrMn, we examined the reorientation effect at different element-specific photon energies. For each photon energy, a new area on the sample that has not been scanned or imaged before is used to eliminate the hysteretic effect of scanning sequence (for details, see “Methods”). During the X-ray illumination, a positive external magnetic field is applied. The corresponding bright area percentage *P*_B_ = *S*(*M*_↑_)/*S*(*M*_↑_ + *M*_↓_) is used to quantify the strength of the reorientation effect. Here *S*(*M*_↑_) and *S*(*M*_↓_) represent the area for magnetization point up (bright) and down (dark), respectively. *P*_B_ as a function of the photon energy is plotted in Fig. 2. When the photon energy is at around Mn L_3_ absorption edge (641.0 eV), a dramatic change of *P*_B_ is observed, while there is no obvious change of *P*_B_ at around Co L_3_ absorption edge (780.4 eV). These results indicate that the exchange bias reorientation is due to the interaction between the soft X-ray photon and the Mn atom and further demonstrate the X-ray-induced manipulation of the antiferromagnetic ordering of IrMn layer.

There are two possible mechanisms of the exchange bias reorientation effect in IrMn layer. One may intuitively attribute this phenomenon to an incoherent heating effect. If the heating effect plays a major role, *P*_B_ should be closely related to the X-ray flux, which is an important factor in determining the X-ray-induced heating^27^. However, it is found that *P*_B_ is proportional to the total photon number and nearly independent of the X-ray flux at a fixed photon number (Supplementary Note 5 and Supplementary Fig. 11). Moreover, the exchange bias reorientation effect gets even stronger at low temperature (see Fig. 2c), further eliminating the major role of heating effect (see more details in Supplementary Note 5). The calculated possible maximum increase in temperature due to the X-ray illumination is only about 0.3 K (the temperature was estimated by COMSOL simulations, for details see Supplementary Note 6), which is negligible compared with the measured blocking temperature (*T*_B_ ≈ 400 K, Supplementary Fig. 4). Therefore, the observed phenomenon is not a thermal effect and different from the previously studied laser-induced thermal modification of the antiferromagnetic order in antiferromagnet/ferromagnet bilayers^28^. Alternatively, coherent photon-induced electron excitation in the antiferromagnetic oxide can cause the reconstruction of antiferromagnetic order^29^ or the change of anisotropy^30,31^. It is plausible here that the X-ray-induced excitation of electrons from 2p to 3d may lead to similar effects. Moreover, the bright area percentage *P*_B_ is independent of the X-ray polarization (Supplementary Fig. 12), further indicating the major role of photon absorption^15^. The correlation between the exchange bias reorientation effect and the photon absorption at Mn L_3_ edge (Fig. 2) shows strong evidence of the X-ray-induced manipulation of antiferromagnetic order. This exchange bias reorientation effect could enable the creation of single skyrmions, which will be discussed in the following section.

### X-ray-induced single skyrmion creation

**Fig. 3:**
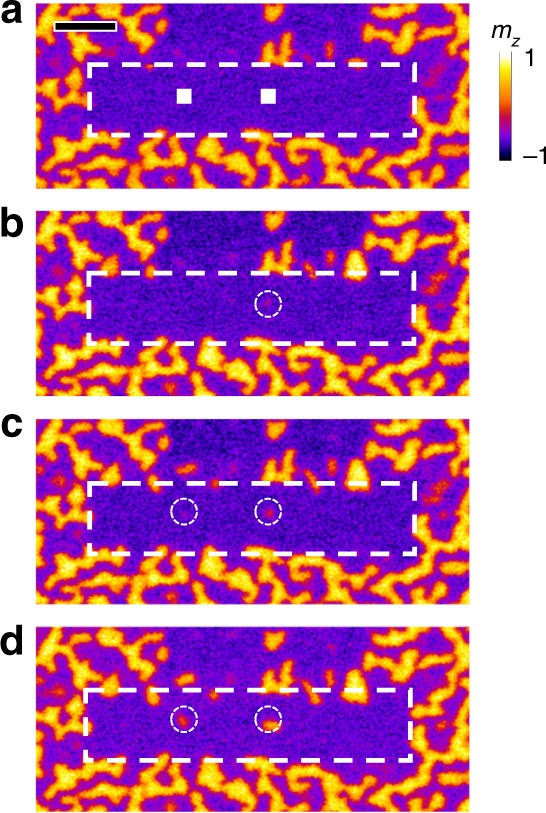
X-ray-induced single skyrmion creation. **a** Magnetic domain pattern (obtained by STXM) after introducing a uniform exchange bias in the middle channel (dashed area, 1 × 7 μm^2^). The two squares indicate the areas of 200 × 200 nm^2^ scanned by using X-rays for creating two individual skyrmions at zero field. **b**–**d** The right skyrmion is first created and then the left one is created. During the reading process, i.e., a low-flux X-ray illumination, the previously created skyrmion (the right one) increases its size (**c**). The size of these two skyrmions can be further increased by performing another reading process (**d**). The white circles have a diameter of 400 nm. The scale bar in **a** is 1 μm. The color represents the out-of-plane component of the magnetization (*m*_*z*_).

In the studied multilayer, skyrmions with a size between 99 and 113 nm can exist under an out-of-plane magnetic field (Supplementary Note 7), indicating that they are energetically stable and can be stabilized via the exchange bias at zero field. The exchange bias reorientation effect furthermore allows the creation of such single skyrmion at zero field. This is done by first creating an exchange-bias-defined 1-μm-wide single domain track as a background (Fig. 3a). Within the track, the exchange bias is reoriented in the negative direction, hence sustaining the single domain of Co layer. To create a skyrmion, the X-ray spot is scanned over a 200-by-200 nm^2^ area (white squares in Fig. 3a). After the illumination, a single skyrmion of around 100 nm diameter can be observed (dashed circle with 400 nm diameter is used to indicate the created skyrmion). The second skyrmion of similar size was also successfully written on the track as shown in Fig. 3c. It can be seen that, while low fluxes were used to image the magnetic film after writing exposures, the additional X-ray illumination still increased the size of the previously created skyrmion (the right one). The size of both skyrmions can be further increased by performing another low-flux reading process (dwell time of 5 ms), as shown in Fig. 3d, which provides us a means to delicately tune the skyrmion size.

**Fig. 4:**
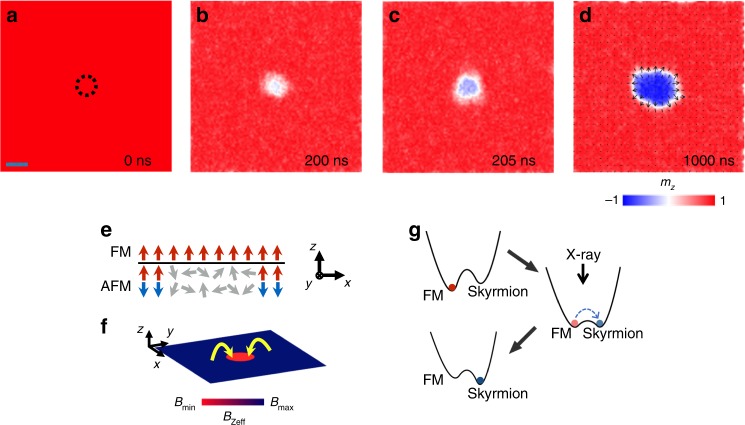
Simulation of the X-ray-induced single skyrmion creation. **a**–**d** Snapshots of the single skyrmion creation process in the *x*–*y* plane. The color represents the averaged *m*_*z*_ across all the magnetic layers. The X-ray illumination within the region enclosed by the dashed line is simulated by turning off the perpendicular external field therein. The spin texture of the created skyrmion is also illustrated in **d**, where the color represents the out-of-plane magnetization and the arrows represent the local magnetization direction. The scale bar in **a** is 44 nm. **e** Schematic of the magnetization in both the ferromagnetic and antiferromagnetic layers during the illumination process. **f** Calculated *z*-component of the effective field during the illumination (before the skyrmion creation happens). The red circle is the X-ray illumination region. The yellow arrows schematically indicate the influence of the effective field. **g** The schematic energy landscape of the ferromagnetic state and skyrmion state before, during, and after the X-ray illumination. The energies were calculated via micromagnetic simulations.

To better understand the skyrmion creation process, we performed micromagnetic simulations to capture its microscopic origin on a full magnetic multilayer stack with 400 nm × 400 nm lateral size and 10 repetitions (for details of the micromagnetic simulations, see “Methods”). An out-of-plane external field is applied to simulate the exchange bias effect. As discussed above, due to the interaction between the X-rays and IrMn, the exchange bias within the X-ray illumination region is destroyed during the illumination process, as shown in Fig. 4e. In the simulations, a circular area with 44 nm diameter is employed as the X-ray illumination region (Fig. 4a). The effect of X-ray illumination is simulated by turning the external field off in the illuminated region.

The simulation results, shown in Fig. 4a–d, demonstrate that a single skyrmion can be successfully created at the X-ray illumination region. This skyrmion creation process can be understood by the calculated energies of the ferromagnetic state and the skyrmion state (Fig. 4g). Before applying the X-rays, the ferromagnetic state has lower energy than the skyrmion state (Fig. 4g top) and thus the skyrmion cannot be created spontaneously. During the X-ray illumination, the energy of the skyrmion state is reduced owing to the vanishing local exchange bias (Fig. 4g middle). The effective fields (including the exchange field, the DMI field, the perpendicular magnetic anisotropy field, and the dipole–dipole field) around the illumination region favor the creation of skyrmion locally as shown in Fig. 4f. The thermal fluctuation, in combination with the effective fields, can drive a transition from the ferromagnetic state to the skyrmion state and create a single skyrmion within the illumination region. After the illumination, the local exchange bias recovers and reorients its direction according to the local magnetization direction, and the skyrmion state acquires lower energy than the ferromagnetic state. The recovered exchange bias within the illumination region further enhances the stability of the created skyrmion (Fig. 4g bottom). Furthermore, the created skyrmion shows a Néel-type structure and the calculated topological charge *Q* = 0.95 (this imperfection is due to the discrete lattice model, finite size effects, and thermal fluctuations, for details see Supplementary Note 8) clearly reflects its topological nature.

## Discussion

Our simulation results also suggest that, by optimizing material parameters, a single skyrmion with around 50-nm size, which is comparable with the X-ray spot size (44 nm), can also be created with our method (Supplementary Note 8). Although the currently studied sample only supports 100-nm skyrmions, further material optimizations in conjugation with smaller X-ray spot size could lead to a promising pathway to optically manipulating even smaller skyrmions.

**Fig. 5:**
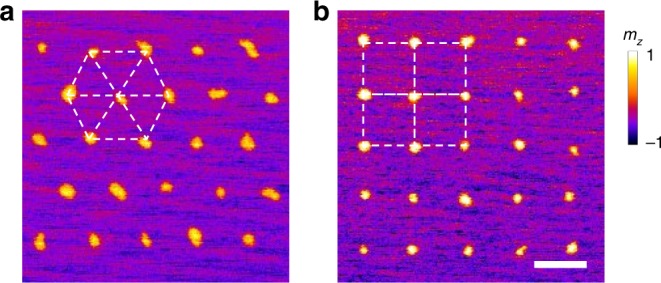
Artificial skyrmion lattice patterned by X-ray illumination. **a** Triangular skyrmion lattice. **b** Square skyrmion lattice. The sample used here consists of Ta (3 nm)/[Pt (1.5 nm)/Co (0.8 nm)/IrMn (5 nm)]_12_/Ta (2 nm). The sample has a uniform exchange bias (induced during the deposition process via an applied external field) and exhibits single domain at zero field. During the creation of the skyrmion lattice, a magnetic field of −200 mT (opposite to the as-grown exchange bias direction) is applied. The scale bar in **b** is 1 μm.

Taking advantage of the precise creation of single skyrmions, artificial skyrmion lattice can also be patterned using X-rays. Such patterned artificial skyrmion lattices with triangular and square arrangements are demonstrated in Fig. 5. Owing to film variations, defects, and other extrinsic factors, it is not that easy to realize fine skyrmion lattice in magnetic multilayers. The X-ray patterned artificial skyrmion lattice thus provides an alternative way to study skyrmion lattice in magnetic multilayers. Moreover, skyrmion lattice with unconventional arrangements can also be patterned, e.g., the square skyrmion lattice shown in Fig. 5b, making such material system a versatile platform for studying numerous topological phenomena, such as the topological Hall effect^2^, topological magnons and their edge states^32,33^, etc.

The modification of an antiferromagnetic order via X-ray illumination allows us for the first time to optically manipulate magnetic order with high spatial resolution. Single skyrmion creation is demonstrated as an example of the application of the local exchange bias reorientation effect. The ultra-high spatial resolution of X-ray can also be used for manipulating other ultra-small magnetic textures. These findings may provide a pathway to imprint a domain into the antiferromagnetic layer through exchange bias effect^34,35^, which might be used for creating an antiferromagnetic skyrmion using X-ray in an exchange bias system^36^. This work will motivate research to study the X-ray manipulation of magnetic orders including ferromagnets and antiferromagnets, which pushes the optical manipulation down to the sub-100-nm scale.

## Methods

### Experimental details

The sample consisting of Pt (5 nm)/Co (0.6 nm)/Ir_22_Mn_78_ (IrMn) (5 nm)/[Pt (2 nm)/Co (0.6 nm)/IrMn (5 nm)]_11_/NiO (2 nm) was grown on silicon nitride membranes and a semi-insulating Si substrate with 100-nm-thick thermally formed SiO_2_ layer by a ULVAC MPS-4000-HC7 magnetic sputtering system. The background vacuum was 4.75 × 10^−6^ Pa. The deposition rate for Pt, Co, and IrMn were 4.18 Å/s, 1.91 Å/s, and 7.77 Å/s at a power of 100 W and a pressure of 0.16 Pa. The deposition rate for NiO was 0.92 Å/s at a power of 100 watts and a pressure of 1.5 Pa. The microscopic images were acquired at scanning transmission X-ray microscope MAXYMUS located at BESSY II synchrotron Helmholtz-Zentrum Berlin (HZB) (Berlin, Germany). The magnetic images were obtained at Co L_3_ absorption edge (780.4 eV) using circularly polarized X-ray light. The X-rays consist of periodic pulses with a width of 50 ps and at the frequency of 499.65 MHz. The beam was focused with Fresnel Zone Plate with outermost zone width ∆*r* = 18 nm that provided a focus spot of 22 nm. The estimated full width of the central peak of the airy shaped focus spot is about 44 nm. The scanning step is 20 nm. A low flux is used for all the imaging purpose and a high flux is used for all the modification of the exchange bias and the creation of single skyrmion. The scanning process can be described as follows: first, (a) the sample was divided into square sub-blocks each with 6 μm × 6 μm lateral size; then (b) the scanning was performed at the central area (3 × 3 μm^2^ lateral area) of the sub-blocks. The photon energy is fixed to be 780.3 eV for all the scanning and imaging processes in Fig. 1. In Fig. 2, different sub-blocks were scanned using different photon energies. For each photon energy, a new sub-block that has not been scanned or imaged before was scanned. After the scanning, an imaging process was done with 780.3 eV photon energy to get the value of *P*_B_.

### Micromagnetic simulations

The micromagnetic simulations were carried out using Mumax3^37^. A magnetic multilayer with ten repetitions was simulated. The geometry of each magnetic layer is 400 nm × 400 nm × 0.6 nm with a mesh size of 4 nm × 4 nm × 0.6 nm. Different magnetic layers are separated by a 7-nm spacer, which is the same as the studied sample. A general Hamiltonian with exchange interaction, DMI, perpendicular magnetic anisotropy (PMA), Zeeman interaction, and magnetic dipole–dipole interaction is considered in the simulations. The following parameters were used in the simulations: the exchange constant *A* = 10 pJ/m, the DMI constant *D* = 1.35 mJ/m^2^, the PMA constant *K*_*u*_ = 950 kJ/m^3^, the saturation magnetization *M*_S_ = 1150 kA/m, and the Gilbert damping constant *α* = 0.4. An out-of-plane external field, *B*_ext_ = 35 mT, is applied to simulate the exchange bias effect of IrMn. A circular region with 44 nm radius is used as the X-ray-illumination region. The simulations were carried out at 300 K with an open boundary condition.

## Supplementary information

Supplementary Information

## Data Availability

The authors declare that the main data supporting the findings of this study are available within the article and its Supplementary Information files. Extra data are available from the corresponding author upon request.
