# Supplementary material for: Creating zero-field skyrmions in exchange-biased multilayers through X-ray illumination
Source: Nat Commun. 2020 Feb 19;11:949. doi: 10.1038/s41467-020-14769-0 (PMC7031520; doi:10.1038/s41467-020-14769-0)
Supplement: Supplementary file 1 — Supplementary Information [file 41467_2020_14769_MOESM1_ESM.docx]

**Creating zero-field skyrmions in exchange-biased multilayers through X-ray illumination**

Yao Guang†^1,2,3^, Iuliia Bykova†^4^, Yizhou Liu†^1,2,3^, Guoqiang Yu^1,2,3^*, Eberhard Goering^4^, Markus Weigand^4^, Joachim Gräfe^4^, Se Kwon Kim^5,6^, Junwei Zhang^7^, Hong Zhang^7^, Zhengren Yan^1,2,3^, Caihua Wan^1,2,3^, Jiafeng Feng^1,2,3^, Xiao Wang^1,2,3^, Chenyang Guo^1,2,3^, Hongxiang Wei^1,2,3^, Yong Peng^7^, Yaroslav Tserkovnyak^5^, Xiufeng Han^1,2,3^ and Gisela Schütz^4^

^1^Beijing National Laboratory for Condensed Matter Physics, Institute of Physics, Chinese Academy of Sciences, Beijing 100190, China

^2^Center of Materials Science and Optoelectronics Engineering, University of Chinese Academy of Sciences, Beijing 100049, China

^3^Songshan Lake Materials Laboratory, Dongguan, Guangdong 523808, China

^4^Max Planck Institute for Intelligent Systems, Heisenbergstraße 3, 70569 Stuttgart, Germany

^5^Department of Physics and Astronomy, University of California, Los Angeles, CA 90095, USA

^6^Department of Physics and Astronomy, University of Missouri, Columbia, Missouri 65211, USA

^7^Key Laboratory for Magnetism and Magnetic Materials of Ministry of Education, Lanzhou University, Lanzhou 730000, People’s Republic of China

†These authors contributed equally to this work.

Email address: [guoqiangyu@iphy.ac.cn](mailto:guoqiangyu@iphy.ac.cn)

**Supplementary Note 1. Characterization of sample’s structure**

Cross-section of the multilayer film was investigated using a HAADF-STEM technique. Fig. 1 shows a representative HAADF-STEM image of the cross-section, clearly showing the layered structure of the multilayer film. To verify the distribution of elements within the multilayer, Energy Dispersive X-ray (EDX) elemental mapping analysis technique was used. The EDX microanalysis (Fig. 2) shows clear oscillations in mapping images of Ni, Mn, Ir, Co, and Pt along the growth direction of the film and has 2 nm Ni element distributing at the top of multilayer.

**Supplementary Figure 1.** The cross-section of the multilayer film captured by TEM.

**Supplementary Figure 2.** Energy dispersive X-ray (EDX) mapping of the elements in the sample.

**Supplementary Note 2. Magnetic characterization measurements**

In this section, we present the measured data for the M-H loop and exchange bias of the samples. We have prepared a series of samples with varying Co thickness: 0.4 nm, 0.6 nm, 0.8 nm, 1.0 nm, and 1.2 nm. The out-of-plane and in-plane M-H measurements are shown in Fig. 3. The samples with relatively thin Co layers show good perpendicular magnetic anisotropy (PMA). The PMA decreases with the Co layer thickness as manifested by the increased out-of-plane saturation field and decreased in-plane saturation field. Also, the exchange bias for these samples after the field-cooling process were characterized. The samples were firstly heated up to 400 K and the then cooled down to room temperature under an out-of-plane field of 1.5 T. Before the filed-cooling process, no obvious exchange bias is observed, as shown in Fig. 4. After the field-cooling process, the exchange bias is characterized at a different temperature, as shown in Fig. 3c. The exchange bias decreases with temperature and Co layer thickness. We have also observed a training effect in the annealed sample (Fig. 5), which is common in exchange bias system.


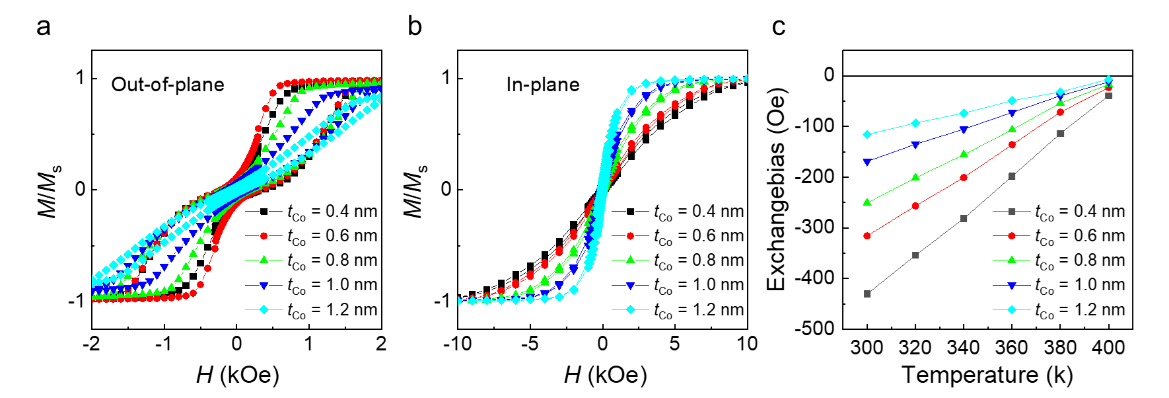


**Supplementary Figure 3.** Out-of-plane (a) and in-plane (b) hysteresis loops for the Pt(5 nm) / Co(*t*_Co_) / IrMn(5 nm) / [Pt(2 nm) / Co(*t*_Co_) /IrMn (5 nm)]_11_ / NiO(2 nm) sample with *t*_Co_ = 0.4, 0.6, 0.8, 1.0, and 1.2 nm. (c) Exchange bias as a function of temperature for the samples with different Co thickness after the field-cooling process. For field cooling process, the sample temperature is increased to 400 K and then lowered down to room temperature under an out-of-plane magnetic field of 1.5 T.


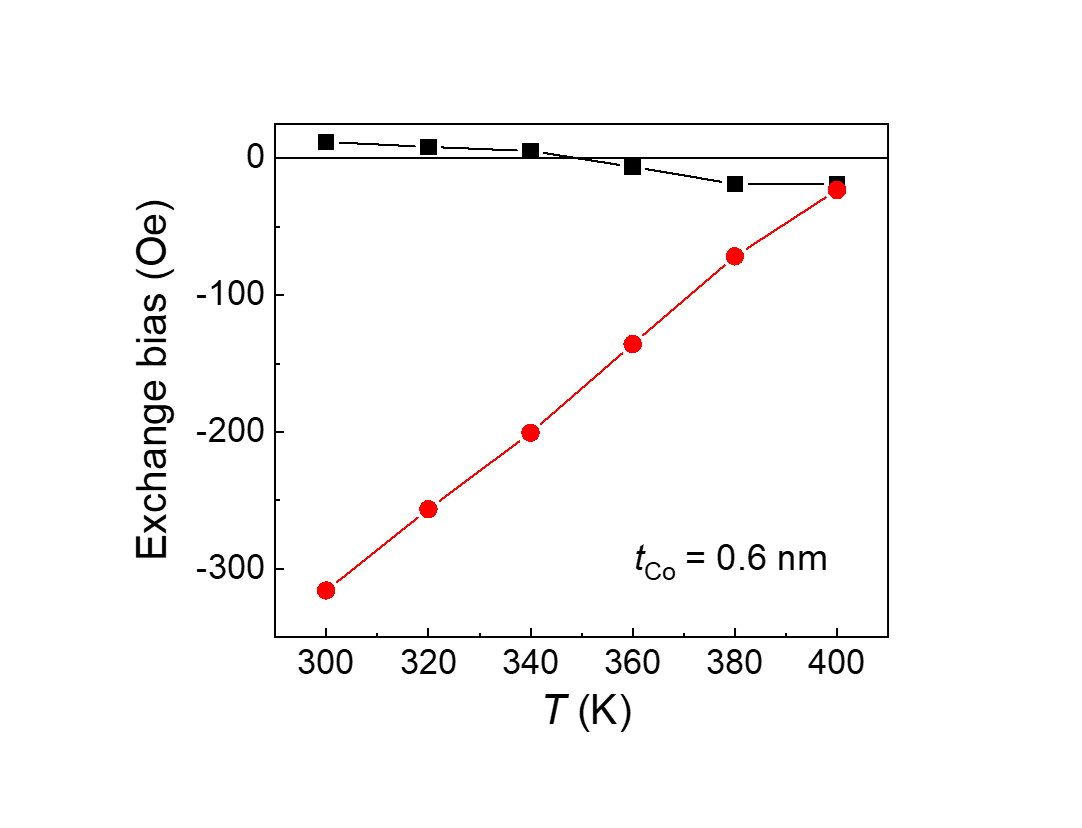


**Supplementary Figure 4.** Exchange bias as a function of temperature for the as-grown sample (black squares) and the sample after a field cooling process (red dots). For field cooling process, the sample temperature is increased to 400 K and then lowered down to room temperature under an out-of-plane magnetic field of 1.5 T.


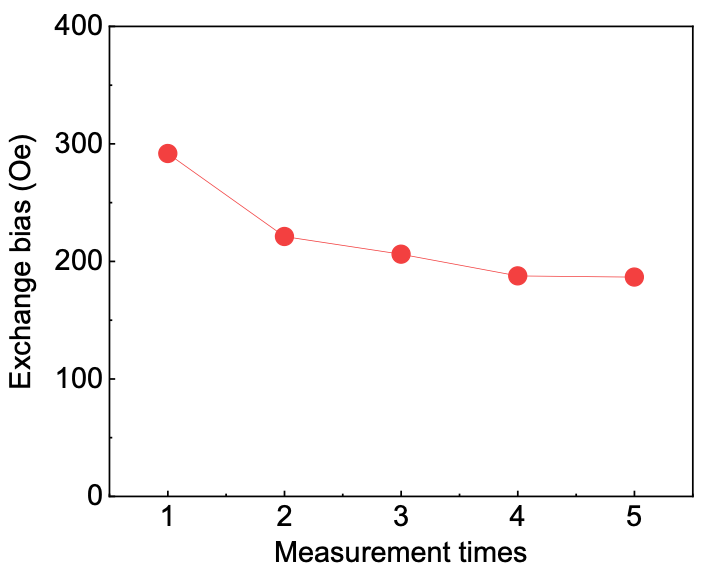


**Supplementary Figure 5.** Training effect of the exchange bias measured in the annealed sample with *t*_Co_ = 0.6 nm.

**Supplementary Note 3. Characterization of the local exchange bias**

In order to prove that a uniform exchange bias is introduced after the X-ray illumination, we measured the local hysteresis loop in a 6 *μ*m by 6 *μ*m region. The central 3 *μ*m by 3 *μ*m area is first illuminated under a magnetic field of −2000 Oe. The curves in Fig. 6a are plotted based on a series of STXM images taken at different field, which is swept from −2000 Oe to 2000 Oe and then back to −2000 Oe. The net magnetization is calculated based on the images for the central 3 *μ*m by 3 *μ*m area (red, with uniform exchange bias) and the surrounding area (black, with randomly distributed exchange bias). Since a negative field is applied during the illumination, a shift of the hysteresis loop towards positive direction is expected (indicating a negative exchange bias). In consistent with this expectation, a clear positive shift of the M-H loop is observed in Fig. 6b, indicating a uniform exchange bias (in negative field direction) is introduced after the X-ray illumination. However, the M-H loop for the surrounding area is still centered at zero field, indicating the absence of uniform exchange bias.

It might be noted that only one-half loop of the central area is shifted in comparison to the loop of the surrounding area. This is because the exchange biases are randomly distributed with ~50% pointing to the positive field direction and ~50% pointing to the negative field direction in the as-grown sample. Thus, the hysteresis loop shows a wasp-waisted shape (black squares in Fig. 6a) for the surrounding area that has not been illuminated by X-ray. For the central area that has been illuminated by the X-ray under an out-of-plane magnetic field, only the exchange bias with direction opposite to the field direction is modified. Therefore, only half of the hysteresis loop is mostly shifted after the illumination (red circles in Fig. 6a), but the whole loop is indeed shifted like the case for the annealed sample (Fig. 6c).

**Supplementary Figure 6.** **a** Measurements of the local hysteresis loop in a 6 *μ*m by 6 *μ*m region. Blue arrows indicate the magnetic field sweep direction. The net magnetization is calculated based on the images for the central 3 *μ*m by 3 *μ*m area (red, with uniform exchange bias) and the surrounding area (black, with randomly distributed exchange bias). **b** Zoomed-in plot for the hysteresis loop of the central area (red circles) in b. The whole hysteresis loop has a shift to the positive direction. **c** Hysteresis loops for the as-grown and annealed samples (replot of the data in Figs. 1b and c). The as-grown sample shows wasp-waisted shaped hysteresis loop, where the top (bottom) half of the hysteresis loop is shifted to the right (left) due to the randomly distributed exchange bias. After annealing, a uniform exchange bias is introduced and only top half of the hysteresis loop is shifted to the negative side due to modification of the exchange bias.

We have also performed other experiments to further prove the existence of a uniform exchange bias and its persistency. In the first experiment as shown in Fig. 7a, positive exchange bias is first induced in both Area I (area enclosed between the large dashed square and small dashed square) and Area II (area enclosed by the small dashed square) through X-ray illumination under a +200 mT external magnetic field. Then, negative exchange bias is introduced in Area II through X-ray illumination under a −200 mT external magnetic field. After setting the magnetic field back to zero from −200 mT, we perform the first imaging process. In the first image, Area II exhibits a single domain with its magnetization pointing along its exchange bias direction (negative direction) while Area I exhibits a multidomain structure. Subsequently, the magnetic field is swept for several cycles as shown in Fig. 7a. Then, another imaging process is performed after setting the magnetic field back to zero from +200 mT. In the second image, while Area II exhibits a multidomain structure, most of the magnetizations in Area I are aligned with its exchange bias direction (positive direction), indicating the persistency of the induced exchange bias. This persistency in combination with the previously measured local hysteresis loop further demonstrate the formation of an exchange bias. It is also noted that there are small parts of the magnetizations in Area I that are not pointed to the positive direction in the second image, which is mainly due to the disturbance from the previous imaging process and the training effect.

It is noted that Area I and Area II with opposite exchange bias cannot maintain in a single domain at the same time. This is can be further understood with Fig. 7b. The hysteresis loop in Fig. 7b has a negative exchange bias as indicated by the red dashed line. However, this exchange bias is not strong enough to shift the loop all the way to the positive direction, so the magnetization distribution of such a hysteresis loop at zero-field will depend on the field-sweeping direction (blue circles in Fig. 8). When the field is sweeping from the same (opposite) direction with the exchange bias, a single-domain (multidomain) state shows up at zero-field. In our experiments, since Area I has an induced positive exchange bias, when sweeping back from a positive (negative) field, it exhibits a single-domain (multidomain) structure. And it is the opposite case for Area II, as shown in the first and second image in Fig. 7. These results further demonstrate that the exchange bias is successfully induced in these two areas. Furthermore, this experiment also demonstrates that the exchange bias reorientation effect is repeatable and independent of the initial exchange bias direction.


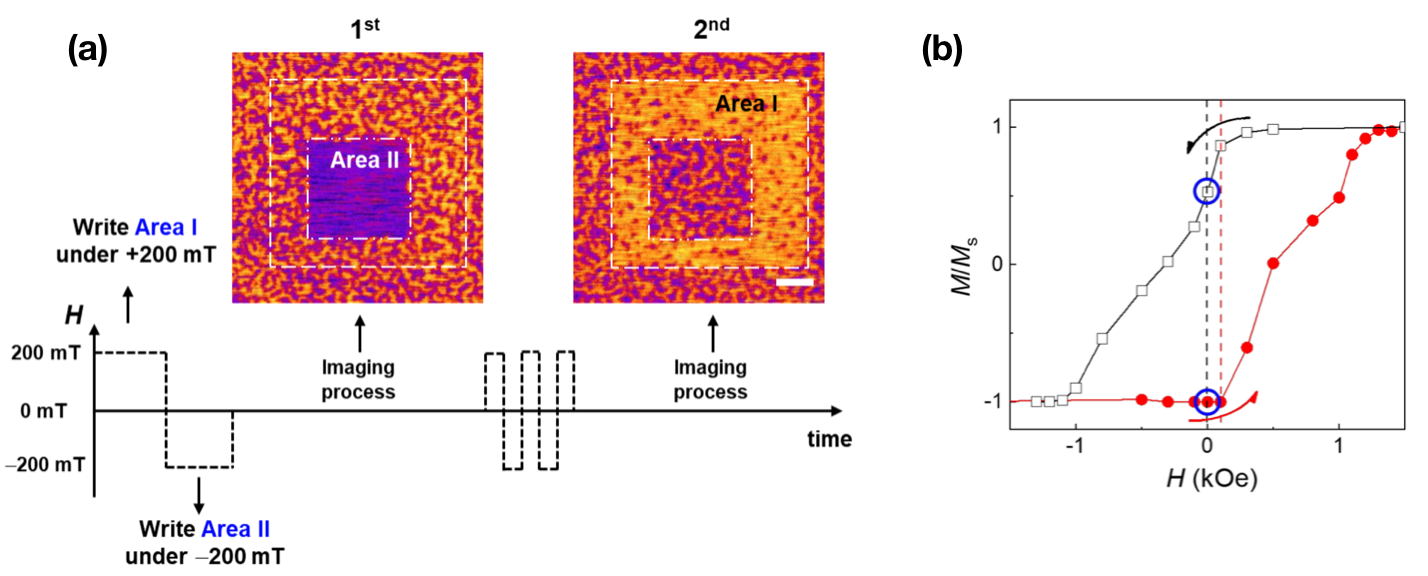


**Supplementary Figure 7.** (**a**) Images of two areas with opposite exchange bias direction. Both Area I and II are first illuminated under a magnetic field of +200 mT and a positive exchange bias is introduced. Then Area II is illuminated under a magnetic field of −200 mT. A negative exchange bias is thus introduced in Area II. The image is captured after decreasing the field to zero. The field is swept between +200 mT and −200 mT for several cycles, and finally set to zero. After the field sweeping, the exchange bias still exists in Areas I and II. The scale bar is 1 μm. (**b**) Measurements of the local hysteresis loop for the area with X-ray-induced exchange bias (from Fig. 6). Red solid dots (black open squares) correspond to the field sweeping from negative (positive) to positive (negative). The blue circles indicate the remanence states.
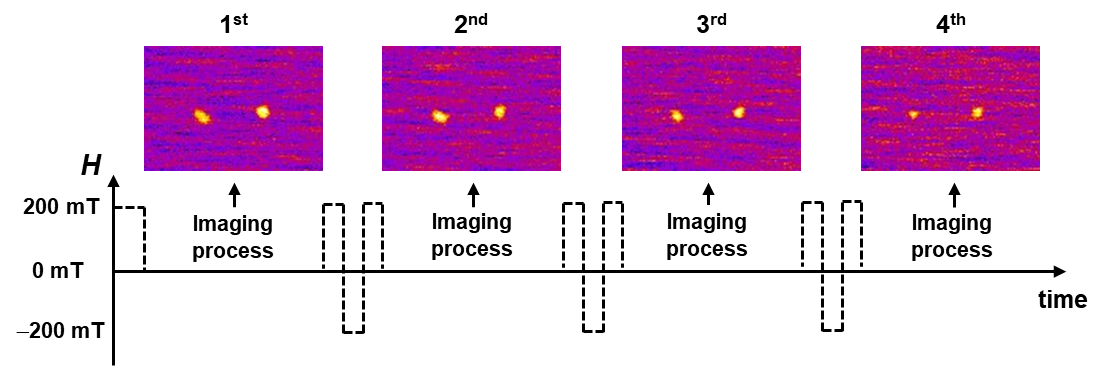


**Supplementary Figure 8.** Images of the induced two skyrmions captured at zero-field after each field cycle. These two skyrmions are still stable after several field cycles. The scale bar is 1 *μ*m.

The second experiment shown in Fig. 8 can prove the modification of the exchange bias from another aspect. A sample consists of Ta(3 nm)/[Pt(1.5 nm)/Co(0.8 nm)/IrMn(5 nm)]_12_/Ta(2 nm) is used in this experiment. During its deposition process, an out-of-plane magnetic field is applied, resulting in a uniform exchange bias in the as-grown sample and thus it exhibits a single domain at zero-field. Then, two skyrmions (see Fig. 8) are introduced through X-ray illumination under an external magnetic field with its direction opposite to the exchange bias. It is also observed that the two skyrmions are persisted after several field cycles. If no exchange bias is modified, a single domain state should still be the ground state at zero-field and the skyrmion cannot exist after several field cycles. The emergence of skyrmions and their persistency also unambiguously proves the modification of the exchange bias.

**Supplementary Note 4. Exchange bias reorientation effect within different samples**

The exchange bias reorientation (EBR) effect occurs in all the studied samples as shown in Fig. 9. It is obvious that the sample with *t*_Co_ = 0.6 nm has the strongest EBR effect. The previous measured M-H loop and exchange bias information provide a hint for this observation. For the sample with *t*_Co_ = 0.6 nm, its exchange bias field is close to its saturation magnetic field, in this case, the exchange bias field can stabilize a single domain. For the sample *t*_Co_ = 1.2 nm, the saturation magnetic field of this sample is much larger than its exchange bias field, so it is difficult to maintain a single domain at zero-field in this sample.

 **Supplementary Figure 9.** Zero-field magnetic domain pattern (6 *μ*m × 6 *μ*m) after illuminating the middle area (3 *μ*m × 3 *μ*m) using a circular polarized X-ray under a magnetic field. The sample consists of Pt(5)/Co(*t*_Co_)/IrMn(5)/[Pt(2)/Co(*t*_Co_)/IrMn(5)]_11_/NiO(2) (thickness in nm). The applied out-of-plane magnetic field is 2000 Oe for the samples with *t*_Co_ = 0.4 nm, 0.6 nm, 0.8 nm, and 1.2 nm. The applied out-of-plane magnetic field is −2000 Oe for the sample with *t*_Co_ =1.0 nm. The bright and dark colors indicate the magnetization of Co pointing in the positive and negative directions, respectively. The color represents the out-of-plane component of the magnetization (*m*_z_).

**Supplementary Note 5. Characterization of the exchange bias reorientation effect as function of temperature, X-ray dwell time, X-ray photon flux, and X-ray polarizations**

In this section, we present additional experimental results to further clarify the mechanisms behind the X-ray induced EBR effect. It should be noticed that all the experiments in this section were performed *in the presence* of a 2000 Oe out-of-plane external field except for the imaging. First, we perform additional tests with different pulse dwell time and fixed photon flux. The observed effect is again quantified by *P_B_* = S(*M*_↑_) / S(*M*_↑_ + *M*_↓_) as defined in the main text and *P_B_* is initially ~ 0.5 as expected for a labyrinth domain pattern. Fig. 10 shows that *P_B_* increases with increasing dwell time at fixed flux. Other than the dwell time, we have also checked the relation between *P_B_* and photon flux at a fixed total photon number. For each flux value, the photon number is fixed by carefully tuning the corresponding dwell time and the result is shown in Fig. 11. From the results shown in Fig. 10 and Fig. 11, it is obvious that (i) *P_B_* is nearly independent of the photon flux at fixed photon number and (ii) *P_B_* is directly proportional to the total photon number. However, if the heating effect is dominant, *P_B_* should be closely related to the photon flux, which is an important factor in determining the X-ray induced heating. Furthermore, as shown in Fig. 4, the blocking temperature of IrMn (approximately its *T_N_*) is around 400 K. This blocking temperature is two orders of magnitude higher than our conservative estimations on the X-ray induced heating effect. These results could help us ruling out the major role of heating effect in the X-ray induced EBR effect observed in the main text.

**Supplementary Figure 10.** *P_B_* as a function of pulse dwell time (duration) at a fixed flux.

*
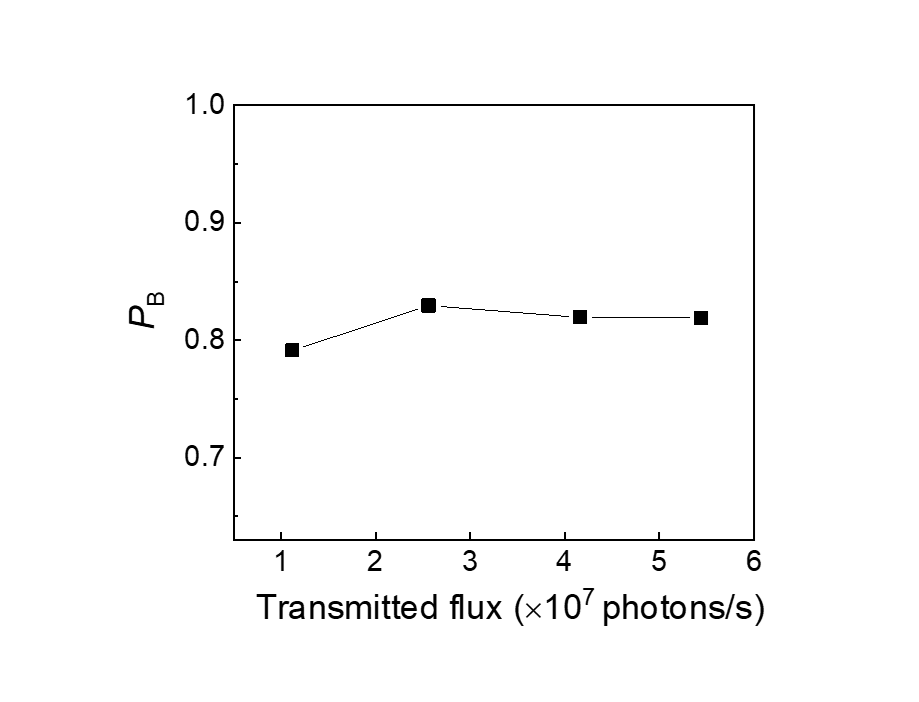
*

**Supplementary Figure 11.** *P_B_* as a function of transmitted flux at a fixed total photon number.

We have also examined the relationship between the EBR effect and X-ray polarization. As shown in Fig. 12, the EBR effect is independent of the X-ray polarization. This independence further evidences that the photon absorption in IrMn layer might be the origin of the EBR effect.

**Supplementary Figure 12.** Percentage of the bright area after the X-ray illumination with positive circular polarization (P), negative circular polarization (N), and linear polarization (L). The X-ray-induced exchange bias reorientation effect is independent on its polarization. The Co layer thickness is 0.4 nm.

Other than the room-temperature results, we have further performed low-temperature measurements of the X-ray induced exchange bias reorientation effect at 105 K and 200 K, respectively. If local heating plays a major role here, then lowering the temperature should greatly inhibit the exchange bias reorientation effect. In contrast, the effect becomes even stronger at low-temperature (Fig. 13), which directly *eliminates* the major role of local heating.

**Supplementary Figure 13.** Zero-field magnetic domain pattern (6 *μ*m × 6 *μ*m) after scanning the central area (3 *μ*m × 3 *μ*m) using X-ray at 300 K, 200 K, and 105 K. Different dwell time is used for scanning each pixel. The applied magnetic field during the scanning process is 2000 Oe. It is clear that the exchange bias reorientation effect is even pronounced at lower temperature. The scale bar is 1 *μ*m.

**Supplementary Note 6. X-ray related membrane heating**

In this section, we estimate the effects of soft X-ray related heating phenomena. We first estimated the total dissipated power at 700 eV. In this case, we could obtain all the non-resonant absorption constants using tabulated Henke table values ^[1, 2]^. In Table 1 all related layer parameters are presented.

**Supplementary Table 1**: Absorption parameters

| material | effective thickness d  [nm] | attenuation length at 700 eV  [*µ*m] ^[2]^ | Absorption coefficient µ at 700 eV  (1/*µ*m] | µd | exp(−*µ*d) |
| --- | --- | --- | --- | --- | --- |
| Pt | 27.0 | 0.055 | 18.182 | 0.491 | 0.612 |
| Co | 7.2 | 0.467 | 2.141 | 0.015 | 0.985 |
| IrMn | 60.0 | 0.066 | 15.152 | 0.909 | 0.403 |
| NiO | 2.0 | 0.317 | 3.155 | 0.006 | 0.994 |
| Si_3_N_4_ | 100.0 | 0.494 | 2.024 | 0.202 | 0.817 |
| total damping |  |  |  |  | 0.197 |

The damping factor of the whole sample stack, including total absorption at 700 eV is therefore 0.197, or in other words, nearly 80% of the photon beam is absorbed by the sample. At 700 eV the beamline provides about 3.7 × 10^8^ photons/sec, where 3.0 × 10^8^ photons/sec are absorbed by the sample. As every 700 eV photon has an energy of 1.12 × 10^−16^ J the total average heating power is 3.32 × 10^−8^ W, which is quite small and about 5 × 10^4^ times less than shown in a similar discussion used in the hard X-ray range ^[3]^.

To estimate the maximum power present during the illumination while the electron bunch in the synchrotron passes the undulator, we assume a typical bunchlength of 50 ps. As we have 5 × 10^8^ bunches/sec the peak power estimate is 1.33 × 10^−6^ W. In our case, the beam at MAXYMUS had a typical FWHM of 22 nm at 700 eV. This gives the related power density of 3.5 × 10^9^ W/m^2^. The illumination times used for the STXM scans were in the range from 5 - 25 ms providing an energy stored for each pixel in the range of 160 - 830 pJ, which is already quite small.

We first estimate the temperature increase for each pulse without considering any thermal conductivity. The volume is the product of the total sample thickness and the area of spot size. We can obtain an effective thermal capacity (using data for each layer) of about 1.2 × 10^−16^ J/K. As each pulse has the energy of about 6.7 × 10^−17^ J, the temperature increase can be calculated to be 0.56 K.

To get a more accurate temperature estimation, we performed a time-dependent and a static COMSOL Multiphysics simulation of the whole stack. We simulated a circular disc with 2 µm diameter of a SiN membrane covered with single layers as given in Table 1 and shown in Figure 14. The heat source of 22 nm diameter has been put in the center of the disc using a continuous power for the static simulations as calculated for the average (peak) power of 3.32 × 10^−8^ W (1.33 × 10^−6^ W) respectively. The rim of the disc has been held at room temperature (293.15 K). Tabulated values for specific heat and thermal conductivity have been used. We also neglected parts of the energy that are ejected by high and low energy electrons (total electron yield) and emitted fluorescence light. Results for upper limit calculation under a continuous X-ray illumination using the peak power are shown in the right part of Figure 14. The absolute maximum temperature increase is only about 0.46 K using the peak power as a continuous average, while the temperature increase for the real average power is only 0.01 K. The left part of Figure 14 shows the temperature of the central spot during the illumination time of 50 ps, and the following decrease in temperature when the beam is off. The observed dynamic increase in temperature is only 0.3 K and therefore negligible for our purpose.

Now we compare our results with the results presented in Ref. ^[3]^. In this reference they had about 3-4 orders of magnitude higher photon flux with about 1.5 orders of magnitude higher photon energy. Regarding thickness, the sample geometry is about to be comparable, but the beam size was 100 nm, and therefore the illuminated area is about 20 times reduced. These authors find a steady state increase of about 8 K and a peak temperature increase of about 20 K. The peak power in the reference could be estimated to be about 50 mW, which is about four orders of magnitude higher. If we take the reduced power and the smaller beam size into account, we could roughly expect a temperature increase during the interaction with the light in our case of 0.1 K, which is in the same order as our precise COMSOL simulation, and therefore demonstrating at least consistency.

As the whole layer stack is already absorbing 80%, an increase in absorption, for example by moving to a resonant absorption line, could only increase the total absorbed power by about one fifth compared to the calculation presented here. Also moving to the Co L_3_ edge is just increasing the power by a small change of the intensity and the higher photon energy of about 778 eV. Therefore, we can conclude that the beam heating of our sample is negligible, by any means.

| 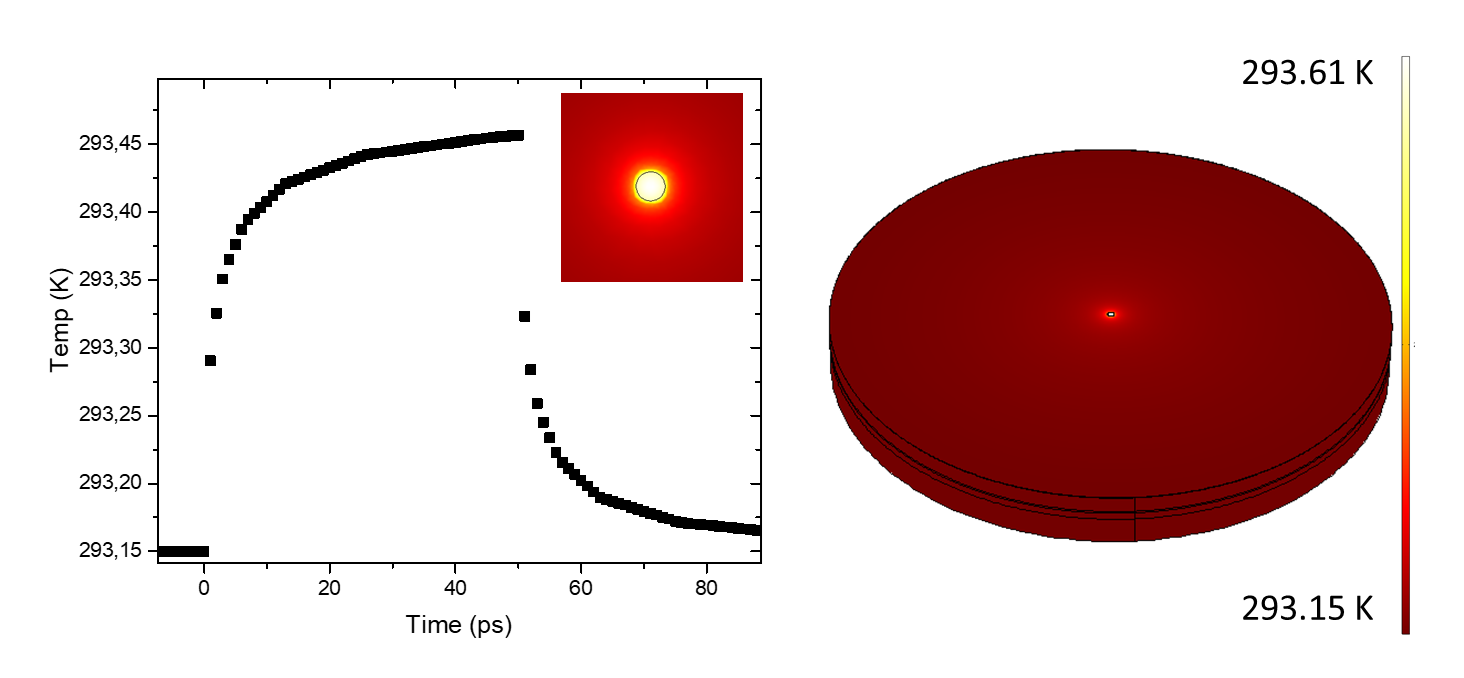 |
| --- |
| **Supplementary Figure 14.** On the right the 2 *µ*m disc is shown, with the 22 nm illumination spot in the center using the peak power as an average. On the left the time-dependent temperature at the rim of the center peak (shown as the black circle in the yellow hot spot of the inset. Legend gives the temperature related color scheme of the high-power static solution. |

**Supplementary Note 7. Magnetic skyrmions resolved via STXM and LTEM**

In this section, we first present the observations of skyrmions stabilized in our sample with STXM, as shown in Fig. 15. The as-grown sample is scanned at different out-of-plane magnetic fields. At zero magnetic fields, the sample shows a labyrinthine domain pattern. As the field increases to 300 Oe, stable skyrmions begin to emerge and coexist with the strip domains. More skyrmions appear, and only few strip domains exist at 600 Oe. At 900 Oe, only skyrmion survives and further increasing the field favors a ferromagnetic state. The skyrmion persists until 1300 Oe. These results demonstrate that single skyrmion is stabilized in the as-grown sample at finite external magnetic fields. So that single skyrmion is also stable with a uniform exchange bias in the same sample, making it possible to create single skyrmion via the EBR effect.


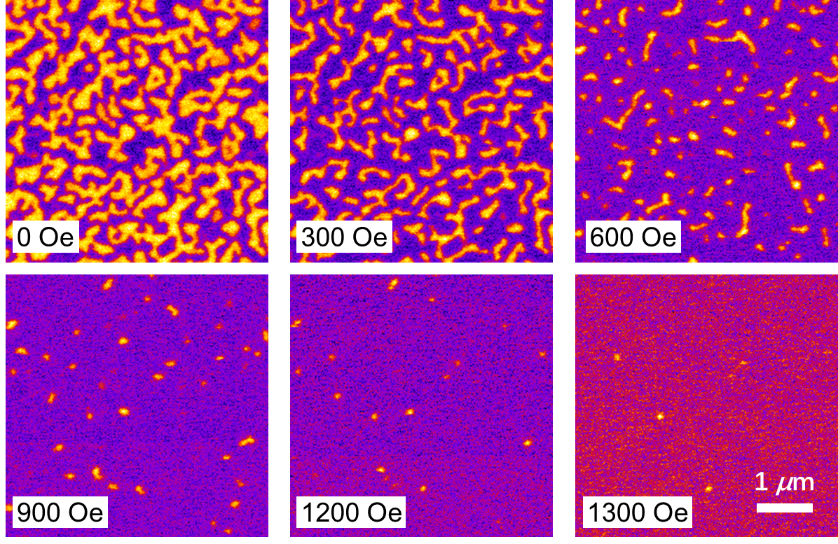


**Supplementary Figure 15.** Evolution of the magnetic domain pattern with an out-of-plane magnetic field in the sample with 0.6 nm-thick Co layer. The scale bar is 1 *μ*m.

To further capture the detailed spin textures observed in our sample, we have performed Lorentz transmission electron microscopy (LTEM) measurements on the studied sample. For the LTEM measurement, the films are grown on the 20 nm-thick Si_3_N_4_ electron transparent membranes. We mainly used the Fresnel method to obverse the skyrmion in a FEI Titan Cubed Themis G2 300. LTEM measurement were carried out at 28º, 0º, and −28º. As shown in Fig. 16, the contrast disappears at 0º and reverses for opposite tilt angles of the sample, which are the features of a Néel-type skyrmion^[4]^.


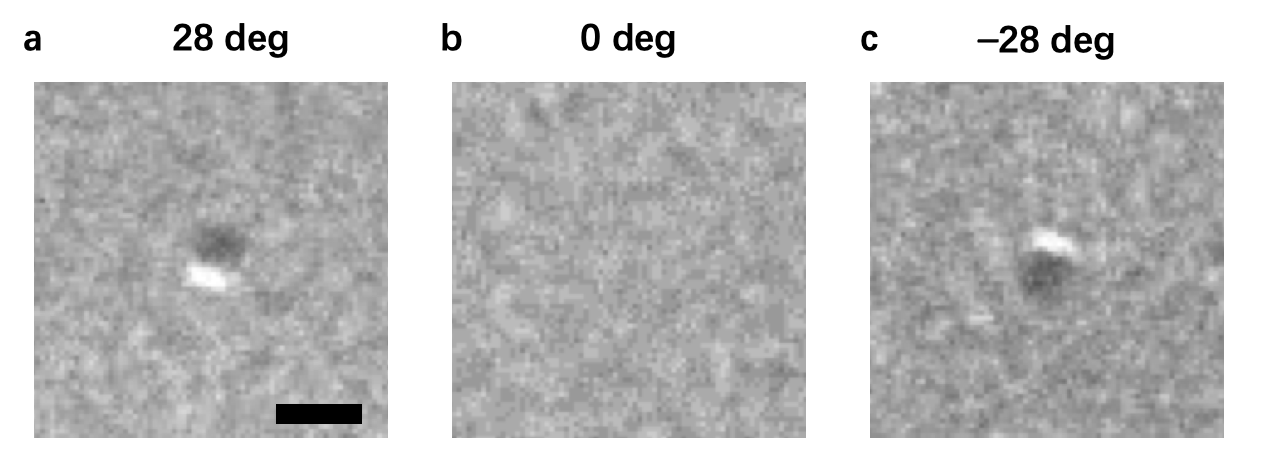


**Supplementary Figure 16.** Skyrmion imaged using Lorentz electron transmission microscopy in the sample with 0.6 nm-thick Co layer. The tilt angles are 28º (a), 0º (b), and −28º (c). The contrast disappears at 0º and reverses for opposite tilt angles (a, c). The scale bar is 500 nm.

**Supplementary Note 8. Micromagnetic simulation details and results of 50-nm-scale skyrmion creation**

In general, skyrmions emerge in magnetic systems due to the competition between different magnetic interactions including the exchange interaction, the Dzyaloshinskii-Moriya interaction and the Zeeman interaction. These three energy terms together can form a simple skyrmion model. However, to better describe skyrmions in magnetic multilayers, perpendicular magnetic anisotropy and magnetic dipole-dipole interaction have to be added in the model. The random fields due to thermal fluctuations are also necessary for capturing the spin dynamics at finite temperature. Thus, all these interactions were taken into account in our simulation models. Furthermore, a full stacking of magnetic multilayer instead of a single 2D effective layer was employed in our simulations. Ten magnetic multilayers were simulated and coupled through magnetic dipole-dipole interaction. In contrast to the conventional 2D effective model, it has been recently demonstrated that the full 3D model provides a better description of the skyrmion physics in magnetic multilayers^[5]^.

To further characterize the topology of the created skyrmion, the topological charge $Q=\frac{1}{4\pi}\iint\boldsymbol{m\cdot(}\partial_{x}\boldsymbol{m\times}\boldsymbol{\partial}_{\boldsymbol{y}}\mathbf{m)}d^{2}\mathbf{r}$ in the main text was numerically calculated on a discretized lattice^[6, 7]^. For an ideal skyrmion, all its spins cover the unit sphere exactly once and facilitate its unit topological charge ($Q=1$). $Q=0.95$ for our simulation result in Fig. 4d (this imperfection is due to the discrete lattice model, finite size effects and thermal fluctuations), which clearly reflects the topology of a skyrmion.

Since the studied sample only supports skyrmion around 100-nm size, the potential of single skyrmion creation via the EBR effect cannot be fully demonstrated experimentally in our current sample. By using micromagnetic simulations, we also show that single skyrmion with a size comparable with the X-ray spot can also be created. A magnetic multilayer with ten repetitions was simulated. The geometry of each magnetic layer is 1 *μ*m × 1 *μ*m × 0.6 nm with a mesh size of 4 nm × 4 nm × 0.6 nm. Different magnetic layers are separated by a 5 nm spacer. The following parameters were used in the simulations: the exchange constant *A* = 10 pJ/m, the DMI constant *D* = 1.5 mJ/m^2^, the PMA constant *K_u_* = 950 kJ/m^3^, the saturation magnetization *M_S_* = 1150 kA/m and the Gilbert damping constant α = 0.1. A 70 mT out-of-plane external field is applied to simulate the exchange bias from IrMn. The simulations were carried out at 300 K with an open boundary condition.

The simulation results are shown in Fig. 17. The created skyrmion size is around 50 nm, which is comparable with the 44 nm spot size. The parameters used in the simulation are all accessible in real experiments, thus providing a routine for future material optimizations and optical manipulations of small nano-scale single skyrmion. Furthermore, the X-ray spot size can be further reduced, making it possible to control ultra-small magnetic solitons and domains optically on the scale of a few tens of nanometers.

**Supplementary Figure 17.** Simulated X-ray induced single skyrmion creation process. The skyrmion size is around 50 nm. **a-d**, Snapshots of the single skyrmion creation process in the *x-y* plane. The color represents the averaged *m_z_* across all the magnetic layers. The X-ray is applied within the circular region enclosed by the dashed line. The scale bar in (**a**) is 44 nm.

**Supplementary References**

[1]. B. L. Henke, E. M. Gullikson, J. C. Davis, in *Atomic Data and Nuclear Data Tables*. (1993), vol. 54, pp. 181-342.

[2]. B. L. Henke, E. M. Gullikson, J. C. Davis, in *Center of X-ray Optics LBNL*. (2002).

[3]. J. W. Harald Wallander. *Journal of Synchrotron Radiation*, *24*, 925-933 (2017).

[4]. S. D. Pollard, J. A. Garlow, J. W. Yu, Z. Wang, Y. M. Zhu, H. Yang. *Nature Communications*, *8*, 14761 (2017).

[5]. W. Legrand, J. Y. Chauleau, D. Maccariello, N. Reyren, S. Collin, K. Bouzehouane, N. Jaouen, V. Cros, A. Fert. *Science Advances*, *4*, eaat0415 (2018).

[6]. G. Yin, Y. Li, L. Kong, R. Lake, C. Chien, J. Zang. *Physical Review B*, *93*, 174403 (2016).

[7]. W. Koshibae, N. Nagaosa. *Nature Communications*, *5*, 5148 (2014).
